# Are the Mineral Acids Formed in the Mouth?

**Published:** 1871-03

**Authors:** Edward C. Chase

**Affiliations:** Iowa City, Iowa


### "ARE THE MINERAL ACIDS FORMED IN THE MOUTH?"

BY EDWARD C. CHASE, D. D. S., IOWA CITY, IOWA.

In the October number of the REGISTER, 1870, I find an article reviewing and criticising a paper published by me in the May number of the *Missouri Dental Journal*, of 1870, with the above heading.

Now, I have no objection in having anything criticised which I may offer to the dental profession, through the medium of the journals, *provided*, the critic thoroughly understands what he is talking about; but I do most emphatically protest in having my article cut up, sentences taken from here and there, and made to serve an entirely different purpose from which they were originally intended; and most of all do I regret that one who is apparently unfamiliar with the new chemical nomenclature, should copy my chemical formulas—based upon the new nomenclature—and parade them in the columns of a widely circulated dental journal, with the remark that "none of the chemical formulas are correctly given," thereby not only making me appear ridiculous, but utterly ignorant of what I am writing about.

I have no disposition nor desire to enter into a controversy with the writer upon what constitutes a mineral acid. By the general acceptance of the term, all of those acids come under this head, which, when united with some base, form a compound or salt, which is known in common language as a mineral, such as calcium sulphate, potassium nitrate, sodium chloride, etc., and as these salts or minerals are formed by the action of sulphuric, nitric and hydrochloric acid respectively, upon calcium, potassium and sodium, or some of their compounds, they would naturally be classed under the head of mineral acids, to distinguish them from those acids which are formed and occur solely in the organic or vegetable kingdom.

In speaking of the formation of nitric acid, I said, "it is commonly prepared by the action of sulphuric acid upon potassium nitrate, thus: $KO_3 N + H_2 SO_4 = HK SO_4 + HN O_3$" nitric acid.

Also in speaking of the action of nitric acid upon the carbonate of lime, I gave the reaction as follows, which is correct: $CaO_3 C + {}_2HNO_3 = Ca_2 NO_3 + CO_2 + H_2 O$. Imagine my surprise on being told by one so well known and universally respected, as the writer of the article referred to, that "none of these formulas are correctly given." Can it be possible that the author is ignorant of the fact that chemistry has changed within the last ten years; that a new chemical nomenclature has been adopted; that the science has been progressing, and that none but "old fogies" adhere to the old formulas?

In one place in my article, the symbol for sulphuretted hydrogen occurs. I gave it then as I do now. $H_2 S$. This formula he says is incorrect, that it should be HS one atom less of hydrogen.

Now, a man who has studied chemistry within ten years, should know that both formulas are correct—the first represents the number of atoms, the latter the number of equivalents of the different elements in the compound, by all modern writers who have adopted the atomic theory it is written $H_2 S$.

The old symbol for sulphuric acid is, $SO_3 + HO$, but now it is written $H_2 SO_4$, which explains more clearly the nature of the combination. The old symbol for water is HO, that is, one equivalent of hydrogen and one of oxygen, but at the present time it is written $H_2 O$, or *two atoms* of hydrogen and one *atom* of oxygen, or by weight two of hydrogen and sixteen of oxygen, while in the old formula it stands one of hydrogen to eight of oxygen. It will be seen that the proportion is the same, but the formulas different. Both are correct. Now it is very unkind for one to accuse another of ignorance in writing the symbol for water $H_2 O$, when he himself has not kept up with the progress of chemical science, or if he has, fails to show it in his writings. Also the old formula for nitric acid has been changed from $NO_5$ to $HNO_3$ that is one molecule of the pent oxide of nitrogen combined with one molecule of water, gives two molecules of nitric acid, thus: $N_2 O_5 + H_2 O = 2HNO_3$.

Thus it is, most of the formulas used a few years ago, to express the composition of the different chemical combinations have been changed; the equivalent theory has been discarded and the atomic theory adopted, so now instead of saying that the equivalent of oxygen is 8, we say that the atomic weight of oxygen is 16. This is adopted by Fowns in his last edition, the universal text-book for dental and medical students; by Roscoe, the great English chemist, and by Hinrichs and Baker, and others who stand at the head of the profession of chemical science.

To one unacquainted with chemical nomenclature and chemical formulas, it would appear by reading the review by this well known writer, that in treating the subject of the "mineral acids," I was handling something of which I was perfectly ignorant.

I will say, however, for the benefit of those who may not have seen the article in question, and therefore have not examined it for themselves, that I defy any one to point out a single mistake in any of the formulas, or in any of the reactions as published by me in the *Missouri Dental Journal.* All that I desire in this article, is to show to the public that I am right in my chemical formulas, as given in the article under consideration. As for the theory advanced in that paper, that these acids, sulphuric, nitric and hydrochloric, might be formed in the mouth under certain conditions, I leave for each one to accept or reject, as the reasoning by which I draw my conclusions, may seem plausible and consistent or otherwise.

